# Appendix Neuroendocrine Tumor: Retrospective Analysis of 4026 Appendectomy Patients in a Single Center

**DOI:** 10.1155/2020/4030527

**Published:** 2020-09-03

**Authors:** Zulfu Bayhan, Yasin Alper Yildiz, Yesim Akdeniz, Emre Gonullu, Fatih Altintoprak, Baris Mantoglu, Recayi Capoglu, Zeynep Kahyaoglu Akkaya

**Affiliations:** ^1^Sakarya University, Faculty of Medicine, Department of General Surgery, Sakarya, Turkey; ^2^Sakarya University, Research and Educational Hospital, Department of General Surgery, Sakarya, Turkey; ^3^Sakarya University, Research and Educational Hospital, Department of Pathology, Sakarya, Turkey

## Abstract

**Background/Aim:**

Appendix tumors are mostly incidentally identified in patients who were operated with the diagnosis of acute appendicitis. They are detected in approximately 1% of appendectomy specimens. Neuroendocrine tumors (NETs) account for over 50% of appendix neoplasms. NETs appearing in the appendix can cause carcinoid syndrome. In our study, we aimed to retrospectively examine the clinical features of patients who underwent appendectomy with the diagnosis of acute appendicitis and diagnosed with appendix NET in the postoperative period. *Materials/Methods.* The records of 4026 patients who were operated with the diagnosis of acute appendicitis between January 2008 and January 2020 at the Department of General Surgery at the Sakarya University Faculty of Medicine, were evaluated retrospectively. Clinical findings, demographic data, surgical findings, and results of the patients with appendix NET, as a result of histopathology, were examined in detail.

**Results:**

16 of 4026 patients were reported as NET. Nine of the patients were male, and seven were female. The average age was 33 (19–49). Any of the patients had no signs and symptoms of carcinoid syndrome. All tumors were located at the tip of the appendix, and the mean tumor diameter was 0.85 cm (0.3–2.5 cm). As a result of pathology, one patient had mesoappendix and one patient had serosa invasion. Right hemicolectomy was applied to both patients. In other patients, meso, serosa, and lymphatic invasion were not detected. Tumor size was 2.5 cm in one of the patients, 1.5 cm in one, and 1.4 cm in the other, and the others were below 1 cm. In the postoperative follow-up, all the patients were discharged on average 2.71 (2–6 days) days without any complications.

**Conclusion:**

Appendix NETs are mostly asymptomatic and localized in a distal third of the appendix. Symptoms are mostly related to tumor size and distant metastases. Clinical behavior and prognosis can best be predicted by tumor size. Complementary hemicolectomy is recommended for tumors larger than 2 cm and tumors smaller than 1 to 2 cm, such as mesoappendix invasion, positive or uncertain surgical margin, high proliferative rate, and angioinvasion. For tumors whose diameter is less than 1 cm, simple appendectomy alone is sufficient.

## 1. Introduction

Neuroendocrine tumors (NETs) are mainly caused by enterochromaffin cells found in the gastrointestinal tract and bronchopulmonary system. Appendix neuroendocrine tumors are the third most common gastrointestinal neuroendocrine tumor after the small intestine and rectum [1]. As with other intestinal NETs, NETs appearing in the appendix can secrete serotonin and other vasoactive substances that cause carcinoid syndrome.

Appendix neoplasms are uncommon. Appendix tumors are mostly incidentally identified in patients operated for acute appendicitis. They are detected in approximately 1% of appendectomy specimens [2]. Preoperative diagnosis can ordinarily be addressed very infrequently.

Neuroendocrine tumors are the most common tumor of the appendix, and it has been mentioned that they constitute more than 50% of the appendix neoplasms [3]. Appendix NETs are most commonly seen in the 30s and 40s, which is much younger than the average age for other primary malignant appendix neoplasms [4].

ANETs do not present a specific clinical description. They are mostly asymptomatic. In patients undergoing appendectomy with the diagnosis of acute appendicitis, they can be diagnosed incidentally [5]. While simple appendectomy is sufficient in the treatment of ANET in some cases, it requires a right hemicolectomy together with lymphadenectomy following oncological principles in some patients [4].

In our study, we aimed to retrospectively examine the clinical features and review the treatment approaches of patients who underwent appendectomy surgery with the diagnosis of acute appendicitis and diagnosed as appendix NET in the postoperative period.

## 2. Methods

The records of 4026 patients who had gone to surgical intervention with the diagnosis of acute appendicitis between January 2008 and January 2020 at the Department of General Surgery, Sakarya University Faculty of Medicine, were evaluated retrospectively. The records of patients who were reported as appendix neuroendocrine tumors as a result of histopathology were examined in detail in terms of clinical findings, demographic data, surgical findings, and outcome analyzes.

## 3. Results

**Figure 1:**
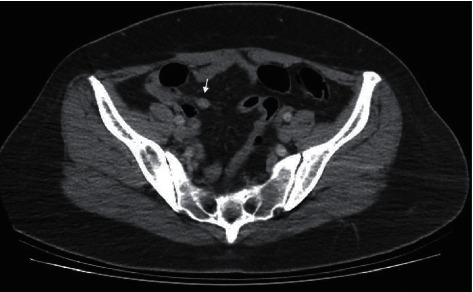
Suspicious mass appearance in the distal of the appendix (white arrow).

**Figure 2:**
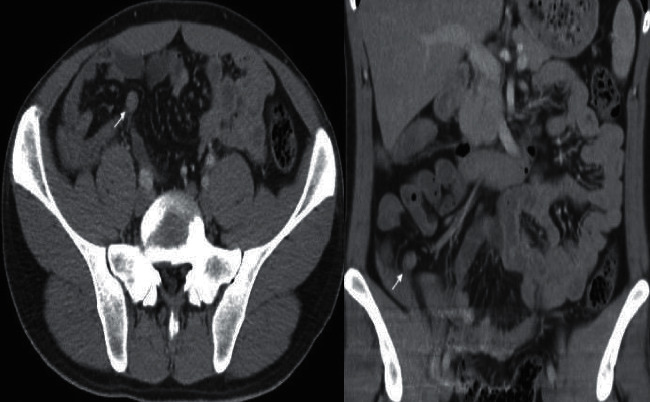
A suspicious mass is seen in the appendix in the axial and coronal CT scan of the same patient in the marked area.

**Figure 3:**
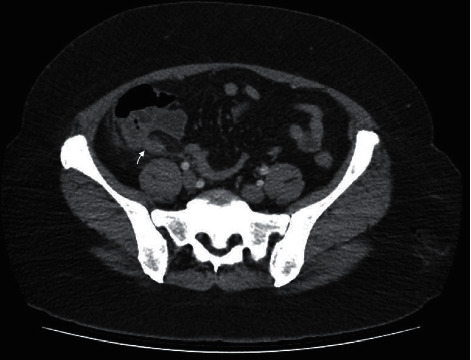
CT findings consistent with the diagnosis of acute appendicitis are observed.

Pathology results of 16 of 4026 patients (0.39%) were reported as NET. The average age of the patients was 33 (19–49), and gender distribution was determined as nine men to seven women. All patients complained of right lower quadrant abdominal pain. Eight patients had anorexia, nausea, and vomiting. The average time between the onset of complaints and admission to the hospital was 2 (1–4) days. None of the patients had additional signs and symptoms such as flushing, diarrhea, asthma, and cyanosis, suggesting carcinoid syndrome. Preoperative leukocytosis was detected in 10 patients. Six patients were diagnosed with appendicitis by abdominal ultrasound (USG). In 6 other patients, appendicitis was not diagnosed with USG, and abdominal computed tomography (CT) was used as an additional imaging method for diagnosis in these patients. Other patients were evaluated as appendicitis only by CT. As an example of our patients who underwent CT scanning, the appearance of suspicious masses in the distal appendix was observed in various CT images specified in Figures 1 and 2. In addition, some of our patients had appearances compatible with acute appendicitis in the CT scan performed in the preoperative period (Figure 3).

**Figure 4:**
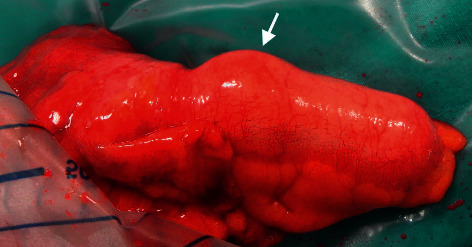
Marked area with suspicion of a mass in the appendectomy material.

One of the patients was operated under spinal, one was epidural, and 14 were operated under general anesthesia. An open appendectomy was performed in 7 patients and laparoscopic appendectomy in 9 patients. None of the patients had appendicitis perforation. Classical McBurney incision was preferred for patients undergoing an open appendectomy. All tumors were located in the distal 1/3 of the appendix, and the mean tumor diameter was measured as 0.85 cm (0.3–2.5 cm range). The area with suspicious mass was marked on the appendectomy specimen of one of our patients and presented as an example (Figure 4).

**Figure 5:**
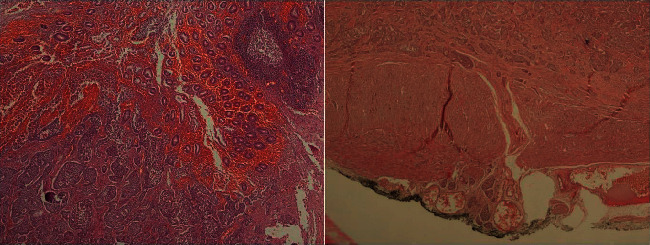
In hematoxylin and eosin sections, tumoral tissue infiltrating the appendix mucosa and submucosa and developing in the insular pattern is observed on the left side of the picture. In the right half of the picture, it is seen that the tumoral tissue infiltrates the submucosa, as well as the muscularis propria and subserosa.

**Figure 6:**
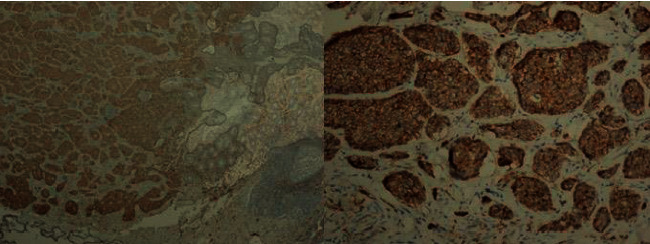
In immunohistochemical examination, CD56 is positive in tumoral tissue.

**Figure 7:**
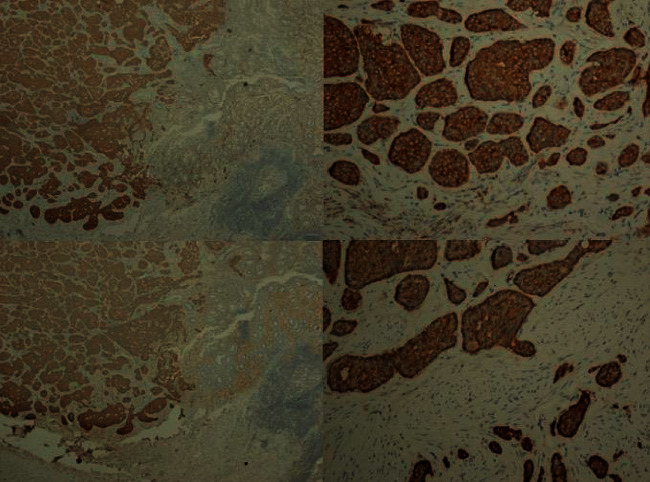
Synaptophysin and chromogranin A are positive in tumor tissue in the immunohistochemical study.

**Figure 8:**
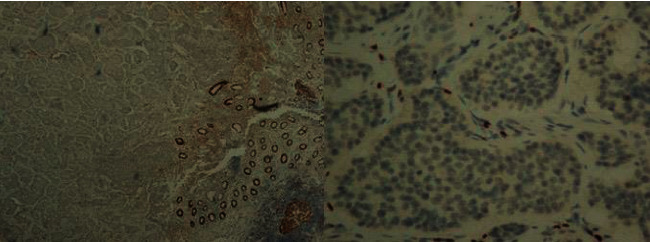
Ki-67 proliferation index is observed to be very low (less than 1%) in tumoral tissue.

As a result of the histopathological evaluation, mesoappendix and serosa invasion were identified in two separate patients. The patient, who had an invasion of the serosa, also had a tumor size of 2.5 cm. Right hemicolectomy was achieved in these two patients. Meso, serosa, and lymphatic invasion were not identified in other patients. In detailed histopathological evaluation, the mitotic rate was below 2 and the Ki-67 index rate was below 3% in all patients. With these results, all patients were determined to have low-grade and well-differentiated NET. The histopathological evaluation images of some of our patients can be seen in the pictures (Figures 5–8).

One of the operated patients was 16 weeks pregnant. During the surgical intervention, oophorectomy was performed on the right ovary due to the exposure of a cystic mass, and the postoperative pregnancy remained uneventful. The pathology result of oophorectomy was reported as a mucinous cystadenoma. In the postoperative follow-up, all patients were discharged on average 2.71 (2–6 days) days without any complications. The demographic and clinical data of the patients are represented in Table 1.

## 4. Discussion

Appendix NETs are seen in 0.2–0.7% of all appendectomies [1]. In our study, the ratio of our patients with appendix NET to all appendectomies was observed at similar rates as in the literature. Most of the appendix NETs are localized in a distal third of the appendix, where it is unlikely to cause obstruction [6]. Most patients are asymptomatic; furthermore, symptoms are mostly related to large tumors and distant metastases. In many patients, diagnosis is made incidentally as a result of histopathological examination of the appendectomy material. Besides, the plasma chromogranin A level, five hydroxyindoleasetic acid levels in 24-hour urine, computed tomography, and octreotide scintigraphy can be applied in diagnosis in patients with suspected NET [7]. In our patients, the diagnosis of ANET after appendectomy with acute appendicitis was made incidentally as a result of histopathological examination of the appendectomy material. Besides, all of the appendix NETs encountered in our patients were localized at the distal end of the appendix.

Approximately 10 percent of appendix NETs are located at the base of the appendix, where they can cause an obstruction and cause appendicitis [6, 8]. In particular, goblet cell carcinoids and poorly differentiated NETs are considered more aggressive [9]. Appendix NETs rarely metastasize to the liver. If liver metastasis or carcinoid syndrome is suspected, the measurement of 5-hydroxy indole acetic acid (5-HIAA), a metabolite of serotonin, is indicated in 24-hour urine [9]. Carcinoid syndrome findings and liver metastasis were not observed in any of our patients.

Clinical behavior and prognosis can best be predicted by tumor size. In approximately 95 percent of patients, the tumor size is less than 2 cm, and these tumors are unlikely to metastasize. In contrast, up to a third of the major lesions are metastatic at diagnosis [10]. However, they often metastasize to their regional nodes rather than the liver. According to the SEER records of NCI, in which 900 patients were analyzed in total, five-year, appendix NET-specific survival rates are as follows: patients with tumor size <3 cm without local nodal or distant metastasis, 100%, patients with tumor size ≥2 but <3 cm with concomitant regional nodal metastasis, or patients with tumor size ≥3 cm without concomitant regional nodal or distant metastasis, 78%. Also, in patients with distant metastatic spread, it was found to be 32 percent. [11]. In addition, the differentiation status and histological grade determined by the rate of mitosis and Ki-67 index provide important information about the clinical behavior of the tumor [12]. In the histopathological evaluation of our patients, it was observed that all of our patients were low-grade and well-differentiated. An important reason for the absence of regional or distant metastases in our patients may be that all of our patients were low-grade and well-differentiated NETs.

The consensus-based guidelines made by the North American Neuroendocrine Tumor Society (NANETS) and the European Neuroendocrine Tumor Society (ENETS), for tumors larger than 2 cm and tumors smaller than 1 to 2 cm, in the presence of deep mesoappendix invasion and in the presence of a positive or uncertain surgical margin, suggest complementary hemicolectomy in the cases of angioinvasion and mixed histology (goblet cell carcinoid, adenocarcinoid) in the case of high proliferative rate (grade 2) [13, 14].

Many clinics agree with these guidelines. However, for tumors smaller than 2 cm, there are also opinions that simply appendectomy is sufficient, even if there is mesoapendix invasion or other adverse histological features. There is a consensus that the simple appendectomy alone is adequate for tumors smaller than 1 cm and in the absence of mesoappendix invasion in NETs between 1.0 and 1.9 cm [4, 15]. All of our patients were operated with an initial diagnosis of acute appendicitis, and an appendectomy procedure was performed. In all of our patients, the diagnosis of appendix NET was made in the postoperative histopathological examination. When the postoperative histopathology results were achieved, it was decided that two of our patients needed complementary right hemicolectomy. Complementary right hemicolectomy was performed in a patient with mesoappendix invasion and in a patient with a 2.5 cm diameter tumoral lesion that showed invasion to the serosa. In addition, due to the advantages of laparoscopic surgery over conventional surgery, we prefer the laparoscopic approach for complementary hemicolectomy operation.

Before the procedure, a complete colonoscopy should be performed to rule out synchronous colon cancer [4, 15]. Perioperative inspection should be performed of the whole intestine because 25 percent of midgut NETs (small intestine and proximal colon) can be multifocal and sometimes associated with other histological types of malignant gastrointestinal tumors [16]. We also performed total colonoscopy before the operation to our patients who underwent hemicolectomy, and no pathology was found in colonoscopy.

In the postoperative follow-up, even the appendix NETs are limited to the appendix and ≤2 cm; they can be managed with simple appendectomy, and follow-up is not required. For more extensive NETs or lymph node-positive tumors, it is recommended that to be treated with right hemicolectomy, within history, physical examination, chromogranin test, and CT imaging of the patient between 3 and 12 months after resection. One year after resection, history, physical examination, and radiographic imaging studies are recommended once every 6 to 12 months with chromogranin test [17].

As a result, appendix NETs are rarely seen. Most of them are detected incidentally. It is necessary to follow the histopathology result after appendectomy. The attention of surgeons is of particular importance in this regard. If NET is determined as a result of the pathology, it is necessary to determine whether additional treatment is required according to the histopathological features of the tumor, following the current consensus-based guidelines.

## Figures and Tables

**Table 1:** Demographic and clinical data of the patients (*n*: 16).

Age	Med	Min	Max
	31	19	49

Sex	Female	Male	
	9	7	

	Mean ± StdDev	Min	Max
Tumor size	9.06 ± 5.1	5 mm	25 mm
Hospitalization time	2 days ± 0.8	1 day	4 days
WBC	11.8 ± 2.6	7.6	16.8

Procedure	Right hemicolectomy	Appendectomy	
	2	14	

Localization	Distal 1/3 of appendix	Stump of appendix	Other
	16	0	0

Perforation	Yes	No	
	0	16	

Type of surgery	Conventional	Laparoscopic	
	7	9	

Anesthesia	Regional	General	
	2	14	

## Data Availability

The data used in the study were obtained from the archives of Sakarya University Medical Faculty, Training and Research Hospital, General Surgery Clinic, after obtaining institutional and ethical approvals.
